# Size-dependent internalization of micro- and nanoplastics induces pro-inflammatory and oxidative stress responses in marine and freshwater fish cell lines

**DOI:** 10.1016/j.cstres.2026.100195

**Published:** 2026-07-08

**Authors:** Kiyun Park, Trang Thi Nguyen, Ihn-Sil Kwak

**Affiliations:** 1 Fisheries Science Institute, Chonnam National University, Yeosu 59626, South Korea; 2 Faculty of Ocean Integrated Science, Chonnam National University, Yeosu 550-749, South Korea; 3 Institute of Animal Medicine, College of Veterinary Medicine, Gyeongsang National University, Jinju, 52828, South Korea

**Keywords:** Plastic contaminants, Cell viability, Inflammation, Immune defense system, Aquatic organism

## Abstract

Microplastics (MPs) are ubiquitously detected in aquatic ecosystems and represent a growing environmental concern due to their persistence, accumulative toxicity, and ability to cross biological barriers, posing substantial risks to fish species. Although numerous studies have investigated the toxicity of MPs in fish, there is limited data on the potential toxic effects of MP exposure at the cellular level. In this study, we aimed to compare the cellular toxicity of polystyrene MPs of different shapes and sizes in fin and muscle cells derived from marine (red sea bream: PMF) and freshwater (fathead minnow: FHM) fish. Our results showed that exposure to MPs of various shapes and sizes did not cause significant changes in cell viability in either cell line. No significant differences were observed, although exposure to spherical MPs (1 μm) induced a decrease in cell viability at relatively high concentrations. The cellular uptake of MPs was observed following exposure to spherical MPs (0.2 and 1 μm), with internalization occurring at 6 h for 1 μm and 24 h for 0.2 μm of incubation. Internalization efficiency was higher for 1 μm MPs than for 0.2 μm MPs. Exposure to spherical MPs (0.2 and 1 μm) induced alterations in intracellular ROS levels and triggered the up-regulation of the *NRF2* gene, a transcription factor involved in the stress response. The expression of pro-inflammatory cytokines, including *TNF-α* and *IL1-β*, was increased by exposure to spherical MPs (0.2 and 1 μm). In addition, this exposure changed the transcriptional responses of immune defense-related genes. Overall, our results suggest that acute exposure to spherical MPs (0.2 and 1 μm) may be related to the disruption of the immune defense system through continuous inflammation at the cellular level in fish, although it does not directly affect cell viability.

## Highlights

• Differential cell viability following exposure to shapes and sizes of MPs on fish cell lines.
• Size-dependent internalization of MNPs in marine and freshwater fish cells.
• Intracellular ROS levels and the up-regulation of the NRF2 gene.
• Induction of the expression of pro-inflammatory cytokines (TNF-α, IL1-β) to MNPs.
• Spherical MPs with 1 μm particles show higher cellular toxicity compared to 0.2 μm particles.

## Background

Microplastics (MPs) have raised global concerns due to their ubiquitous detection, persistence, and potential toxicity in aquatic ecosystems, driven by mass consumption and widespread usage.^1^, ^2^ MP particles originate as fragmented debris from plastic masses in oceanic and freshwater environments, resulting from physical abrasion—caused by wind, waves, and tides—as well as UV exposure.^2^, ^3^ This widespread pollution represents a serious threat to both open ocean and freshwater ecosystems.^4^ MPs found in aquatic environments exhibit a variety of sizes and shapes, including granules, fragments, fibers, flakes, and spheres with a diameter or length of less than 5 mm.^5^ Fibrous MPs are often the most abundant shape, particularly in sediments and water samples, largely originating from synthetic textiles and fishing gear.^6^, ^7^, ^8^ Fragmented MPs are also highly prevalent, especially in river systems, forming when larger plastic items (e.g., bottles or containers) degrade due to UV radiation and mechanical forces.^9^ Although MPs are defined as ranging from 1 µm to 5 mm, the majority of MPs found in environmental samples fall within the smaller size ranges, typically less than 0.5 mm or 200–500 µm. These smaller MPs are difficult to detect in field environments, suggesting that their widespread presence might be underestimated in field sampling.^8^, ^9^, ^10^

Fish play a pivotal role in aquatic ecosystems by promoting nutrient cycling, maintaining water quality, and controlling habitat structure within the food web.^2^, ^11^, ^12^ These species contribute to overall ecosystem stability and productivity, ranging from tiny plankton-eaters to large predators,^13^ and serve as key indicators of ecosystem health.^12^ MPs are ingested by aquatic organisms such as fish; once they enter the body, they induce harmful effects on reproductive and developmental systems through oxidative damage and immune disruption.^7^, ^14^ Fish often ingest MPs through the mouth, mistaking them for food, or through the gills during respiration. MP accumulation leads to physical occlusion, abrasion of internal surfaces, and inflammatory responses in the digestive system.^15^ A stomach full of MPs can induce a false sense of satiation, which is linked to growth retardation due to nutritional deficiencies.^16^, ^17^ Furthermore, small MPs adhering to gill surfaces stimulate excessive mucus production and interfere with gas exchange.^18^ MP exposure also triggers antioxidant defense mechanisms by promoting reactive oxygen species (ROS) generation.^19^, ^20^ Recent studies have shown that MPs and nanoplastics (NPs) cause developmental abnormalities and oxidative damage in zebrafish.^21^, ^22^, ^23^

Fish cell lines are valuable tools for screening the risks posed by contaminants. Recently, toxicity studies of MPs and NPs using these cell lines have been actively conducted as alternatives to animal testing.^24^ In rainbow trout gill cell lines, NP exposure has been shown to induce cellular internalization through energy-dependent endocytic processes.^25^ Additionally, polystyrene NPs increased the activities of alkaline phosphatase and acid phosphatase in fish fibroblasts.^26^ Thus, investigating the effects of these plastic particles on fish cell lines is crucial for comprehensive risk assessment. Fish fins as external exposure is in constant contact with contaminated water. This line is ideal for studying initial physical and chemical damage, outer membrane adhesion, and direct cellular penetration of contaminants or infections.^27^ Muscle as internal accumulation is a major target tissue where ingested or inhaled NPs accumulate via blood circulation. It is perfect for evaluating long-term internal risks like oxidative stress and cellular inflammation.^28^ The present study aims to evaluate the cytotoxicity of MPs and NPs of different sizes, shapes, and concentrations on fin and muscle cells derived from marine (red sea bream: PMF cells) and freshwater (fathead minnow: FHM cells) fish. The specific objectives are to: (1) determine cell viability following exposure to MPs and NPs of different sizes and shapes; (2) observe the internalization into fish cells according to the size and shape of MPs and NPs; and (3) investigate ROS production and the expression patterns of stress-related and cytokine genes induced by MP and NP exposure.

## Materials and methods

### Microplastic and nanoplastic materials

The MPs used in this study consisted of two shapes: spherical particles of three sizes (1, 6.8, and 27–32 µm) and fragments of two sizes (15–20 and 45–75 µm). The NP was spherical with a size of 0.2 µm. Fluoresbrite® (FB) polystyrene microspheres (0.2 μm, Cat. No. 17151–10; 1 μm, Cat. No. 17154) were purchased from Polysciences (Warrington, PA, USA). Fluorescent green polyethylene microspheres (27–32 µm) and yellow spherical MPs (6.8 µm) were purchased from Cospheric LLC (Goleta, CA, USA). Fragmented polyethylene terephthalate MPs (15–20 and 45–75 µm) were supplied by the Korea Institute of Industrial Technology (KITECH, Ansan, Korea).

The surface morphology of the fragmented MPs was characterized using a scanning electron microscope (HR FESEM SU8010; Hitachi, Tokyo, Japan) and an Olympus BX51 optical microscope (Olympus, Tokyo, Japan). The chemical composition of the fragmented MPs was verified by Raman microscopy using 532 and 785 nm lasers (RAMANtouch, Nanophoton, Osaka, Japan). The actual size and size distribution of the fragmented MPs were determined using a fluorescence microscope (Nikon, ECLIPSE Ti–U, Melville, NY, USA) with 515–560 nm excitation and 630 nm emission filters. Additionally, all MPs and NPs were visualized under an FV3000 laser scanning confocal microscope (Olympus).

### Cell culture conditions

The *Pagrus major* fin (PMF) cell line was derived from the fin of the red sea bream (*P major*). The fathead minnow (FHM) cell line, a permanent epithelial cell line, was derived from the muscle of the FHM (*Pimephales promelas*). Both cell lines were cultured at 25 °C in Leibovitz’s L-15 Medium (Cat. No. 11415064; Gibco, CA, USA) supplemented with 10% (v/v) fetal bovine serum (FBS; Cat. No. 16000–044, Gibco) and 1% antibiotic-antimycotic (Cat. No. 15240–062, Gibco).

### Cell viability assay

The cytotoxic effects of MP and NP exposure on PMF and FHM cells were assessed using the MTT assay. Cells were seeded at a density of 3 × 10⁴ cells per well in 96-well plates and incubated for 24 h in L-15 medium containing 10% FBS. The PMF and FHM cells were then incubated with various concentrations (1, 10, 25, 50, 75, 100, and 200 μg/mL) of 0.2 and 1 µm spherical MPs or with concentrations (50, 100, 250, 500, and 1000 μg/mL) of spherical MPs (6.8 and 27–32 µm) and fragmented MPs. After 48 h of exposure, the media were removed, and the cells were washed twice with PBS. The cells were then treated with 5 mg/mL of MTT solution in fresh medium for 4 h at 25 °C in the dark**.** Following incubation, the medium was replaced with 150 µL of DMSO and incubated for 15 min to dissolve the formazan crystals**.** The absorbance was measured at 540 nm using a microplate reader (Mobi; MicroDigital Co., Ltd., Seoul, Korea).

### Immunofluorescence imaging for the observation of MP and NP uptake

PMF and FHM cells were seeded onto glass coverslips and exposed to MPs and NPs after 24 h. The uptake and internalization of MPs and NPs were observed at 6, 24, and 48 h using a confocal microscope. At each time point, the cells were washed three times with PBS and fixed with a fixative solution (Cat. No. FB002; Invitrogen, CA, USA) for 15 min. After another three washes with PBS, the cells were mounted using Fluoromount-G™ Mounting Medium (Cat. No. 0100–01; SouthernBiotech, Birmingham, AL, USA). Fluorescence signals were then captured and analyzed using an FV3000 laser scanning confocal microscope (Olympus). Fluorescent staining was used on MPs and NPs only for cellular uptake experiments in the study.

### Detection of intracellular ROS production

Intracellular oxidative stress levels were measured using the DCFDA/H2DCFDA-Cellular ROS Assay Kit (Cat. No. ab113581; Abcam, Cambridge, UK). Each cell line was seeded at a density of 3 × 10⁴ cells per well in 96-well plates and grown for 24 h in phenol red-free L-15 medium containing 10% FBS. The cells were then incubated with MPs and NPs (200 μg/mL) for 48 h. For positive control (PC), cells were pre-treated with 0.35% of hydrogen peroxide (H_2_O_2)_ for 30 min to induce oxidative stress. According to the manufacturer’s instructions, the fluorescence signals were detected using a Synergy H1 multimode microplate reader (Agilent Technologies, Santa Clara, CA, USA).

### RNA extraction and gene expression analysis using quantitative real-time PCR

To extract total RNA, PMF and FHM cells were incubated for 48 h with 200 μg/mL of MPs and NPs and then washed twice with cold PBS. Cells were collected in 1 mL of RiboEx (GeneAll, Seoul, Korea), and 400 μL of chloroform was added to the homogenate. The mixture was homogenized by intermittent vigorous vortexing for 5 min followed by a 1-min rest period. The homogenate was then centrifuged at 12,000 rpm and 4 °C for 15 min. The aqueous phase was carefully transferred to a new tube and mixed thoroughly with 400 μL of isopropanol for 10 min at room temperature before being transferred to a binding column. The column was centrifuged at 12,000 rpm and 4 °C for 30 min and washed twice with RPE buffer. The column-bound RNA was eluted with nuclease-free water. cDNA was synthesized using the QuantiTect® Reverse Transcription Kit (Qiagen, Hilden, Germany) according to the manufacturer's instructions. Quantitative real-time PCR (qPCR) was performed using GoTaq® qPCR Master Mix (Promega, Fitchburg, WI, USA) on a Bio-Rad CFX Opus 96 real-time PCR system. The reaction mixture (20 μL) consisted of 2 μL of cDNA, 2 μL each of forward and reverse primers, 10 μL of qPCR Master Mix, and 4 μL of nuclease-free water. Relative mRNA expression levels were analyzed using Bio-Rad CFX Maestro software. All PCR produced a strong linear fit with the cDNA template dilutions (R^2^ ≥ 0.95), and the PCR efficiency was ≥93% for assays. The relative mRNA expression level of each transcript was determined using the best reference gene selected (*GAPDH*) as an internal reference gene according to the 2^–ΔΔCt^ method.^29^ The primer sequences used in this study are listed in Supplementary Table 1.

### Statistical analysis

Data are presented as the mean ± standard deviation (SD). Statistical analysis was conducted using an unpaired Student’s *t*-test via GraphPad InStat. Significance levels were denoted as * *P*< .05 and ** *P* < .01, indicating statistically significant differences between the groups.

## Results

### Cell viability to exposure of MPs and NP

**Fig. 1:**
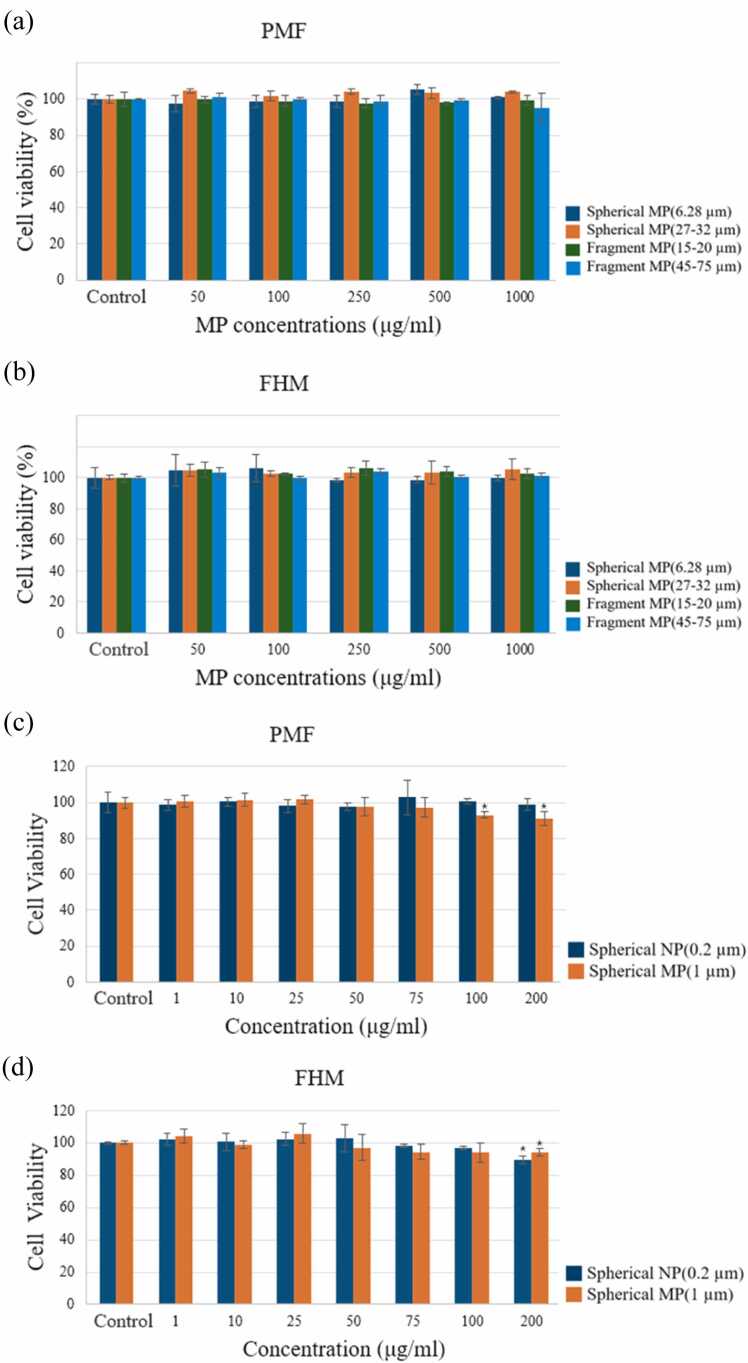
Cytotoxic effects of MPs and NPs of different shapes and sizes on PMF cells (a, c) and FHM cells (b, d) following 48 h of incubation at various concentrations, as determined by the MTT assay. Data are presented as the mean ± SD of triplicate samples from independent experiments. Significant differences from the control group are denoted by asterisks (* *P* < .05).

We investigated the cell viability of PMF and FHM cells following exposure to MPs and NPs of various shapes, sizes, and concentrations (Figure 1). After 48 h of incubation, no significant differences in cell viability were observed in either PMF (Figure 1a) or FHM (Figure 1b) cells across all concentrations of the two spherical MPs (6.8 and 27–32 µm) and two fragmented MPs (15–20 and 45–75 µm) compared to the control groups. However, the viability of PMF cells significantly decreased following exposure to relatively high concentrations (100 and 200 μg/mL) of 1 µm spherical MPs (*P* < .05; Figure 1c). A similar downward trend in PMF cell viability was also observed during long-term exposure (2 weeks) to high concentrations of 1 µm spherical MPs (Supplementary Figure 1). In FHM cells, viability was significantly decreased after exposure to 1 µm spherical MPs and 0.2 µm spherical NPs at the highest concentration (200 μg/mL; *P*< .05; Figure 1d). In both fish cell lines, regardless of the MP shapes (spherical or fragment), only small MP and NP (0.2–1 µm) showed an effect on cell viability.

### Cellular uptake of MPs and NP in fish cell model

**Fig. 2:**
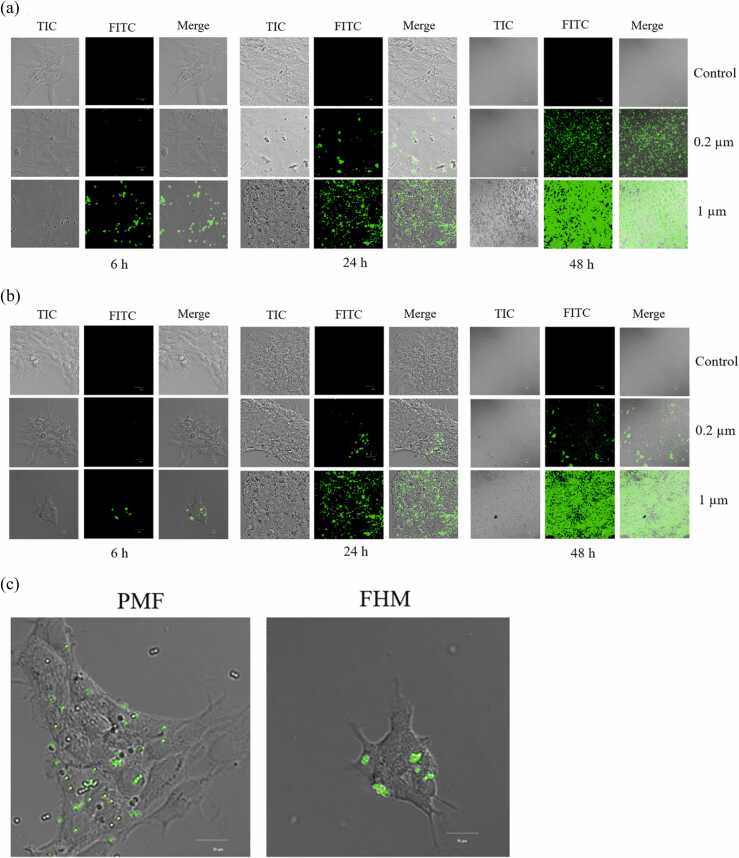
Cellular internalization of MPs and NPs in PMF (a) and FHM (b) cells. Both cell lines were incubated with 25 μg/mL of green fluorescent-labeled 1 µm MPs and 0.2 µm NPs, then visualized under a confocal microscope at 6, 24, and 48 h. (c) Magnified images of individual PMF and FHM cells showing punctate fluorescence patterns.

The cellular uptake of MPs and NPs of different shapes and sizes was investigated in PMF and FHM cells (Figure 2). No cellular uptake signals were detected following exposure to the two spherical MPs (6.8 and 27–32 µm) or the two fragmented MPs (15–20 and 45–75 µm) in either cell line, compared to the controls, which showed no fluorescence (Supplementary Figure 2). However, in both PMF and FHM cells, fluorescence signals were detected for the 1 µm spherical MPs after 6 h of incubation (Figure 2a and b). By 24 h, signals indicating cellular uptake were also observed for the 0.2 µm spherical NPs. The signal intensity increased gradually with incubation time. At 48 h, the fluorescence signals were stronger and more distinct for the 1 µm spherical MPs than for the 0.2 µm spherical NPs in both cell lines (Figure 2a and b). Furthermore, the internalized quantity of 1 µm spherical MPs was higher than that of 0.2 µm spherical NPs (Supplementary Figure 3). The distribution of the fluorescent signals appeared punctate and was often localized at or near the cell surface (Figure 2c), suggesting that MP and NP exposure resulted in strong surface adherence. Notably, no significant changes in cell morphology were observed during incubation with the MPs and NPs. Considering cell viability and intracellular uptake results, the subsequent experiments compared only the results for two small plastics (0.2–1 µm).

### Intracellular ROS production to MP and NP exposures

**Fig. 3:**
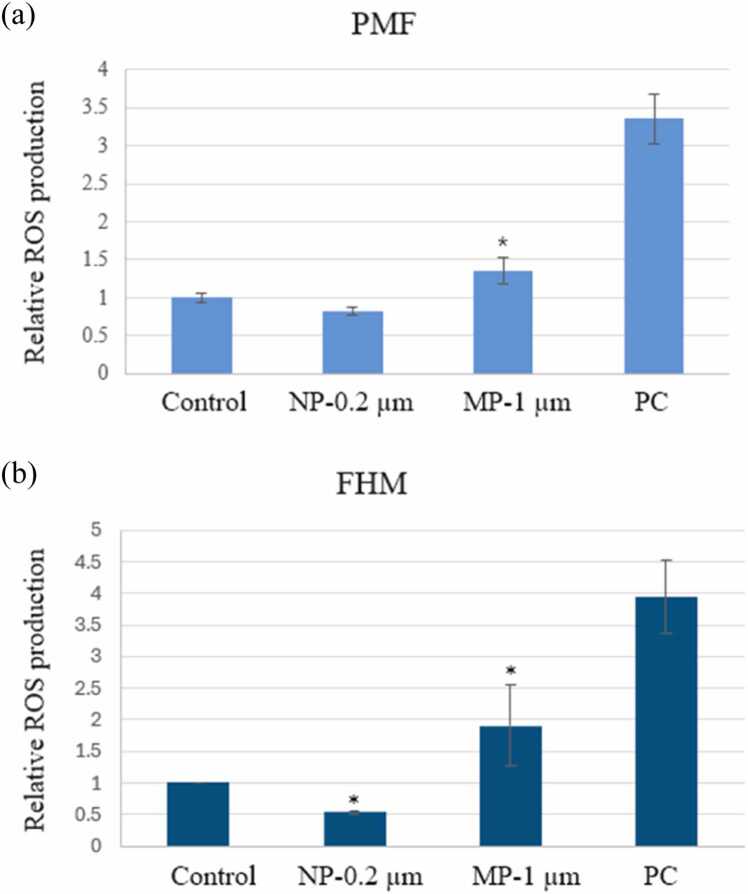
Intracellular ROS production in PMF (a) and FHM (b) cells following exposure to 1 µm MPs and 0.2 µm NPs. Data are presented as the mean ± SD, and significant differences from the control group are denoted by asterisks (* *P*< .05). PC, positive control.

Intracellular ROS production induced by MP and NP exposure was assessed to evaluate oxidative stress in PMF and FHM cells (Figure 3). In PMF cells, ROS production significantly increased following exposure to 1 µm spherical MPs (*p* < 0.05; Figure 3a). Similarly, oxidative stress was detected in FHM cells exposed to 1 µm spherical MPs, whereas ROS production was relatively lower following exposure to 0.2 µm spherical NPs (Figure 3b).

### Oxidative stress-related gene expressions to exposures of MP and NP

**Fig. 4:**
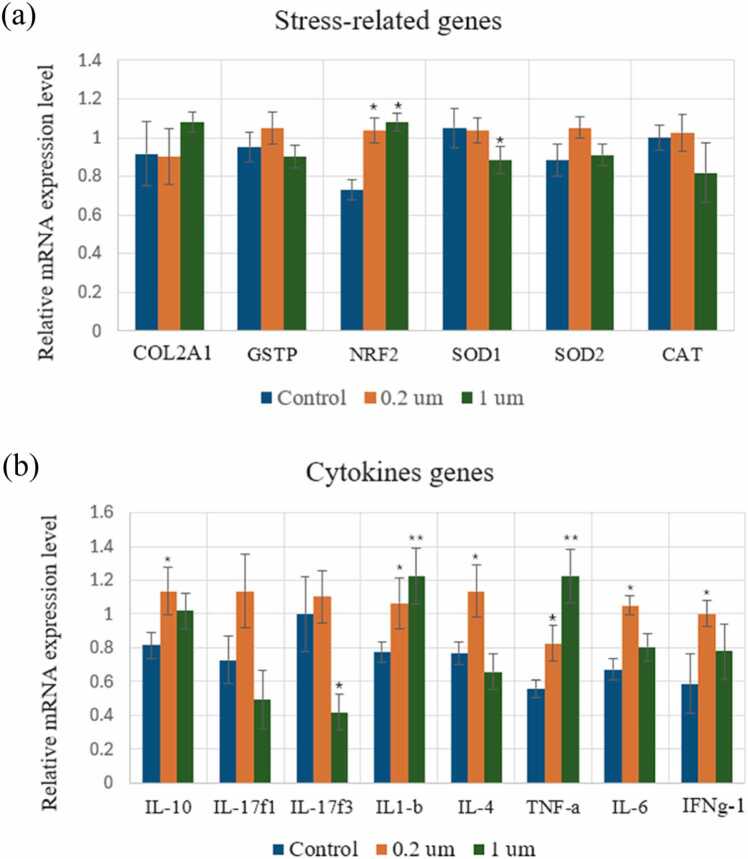
Transcriptional expression of stress-related genes (*COL2A1*, *GSTP*, *NRF2*, *SODs*, and *CAT*) and cytokines (*IL-10*, *IL-17f1*, *IL-17f3*, *IL-1β*, *IL-4*, *TNF-α*, *IL-6*, and *IFN-γ1*) in PMF (a) and FHM (b) cells following exposure to 1 µm MPs and 0.2 µm NPs. Data are presented as the mean ± SD, and significant differences from the control group are denoted by asterisks (* *P* < .05 and ** *P*< .01).

To investigate the effects of MP and NP exposure on oxidative stress, we evaluated the transcriptional expression of key antioxidant genes (*CAT*, *SOD1*, and *SOD2*), oxidative stress and detoxification-related genes (*NRF2* and *GSTP*), and a collagen-related gene (*COL2A1*) in the fish cell models (Figure 4). In FHM cells, the expression of the *NRF2* gene was significantly induced following exposure to 1 µm spherical MPs and 0.2 µm spherical NPs (*P*< .05; Figure 4a). Conversely, the mRNA expression of *SOD1* was significantly decreased in response to 1 µm spherical MPs (*P*< .05). However, no significant responses were observed for other key antioxidant and collagen-related genes in FHM cells following MP and NP exposure.

### Expression of cytokine genes to exposures of MP and NP

To evaluate whether MP and NP exposure affects inflammatory and apoptotic responses, the transcriptional expression of eight cytokine genes was analyzed in the fish cell model (Figure 4b). The expression of *IL-1β* and *TNF-α* was significantly induced following exposure to 1 µm spherical MPs and 0.2 µm spherical NPs in FHM cells (*P* < .05; Figure 4b). The most pronounced upregulation of these genes was observed following exposure to 1 µm spherical MPs (*P*< .01). Additionally, the expression of *IL-10*, *IL-6*, *IL-4*, and *IFN-γ1* was significantly increased in response to 0.2 µm spherical NPs. Although a similar inductive response for these genes was observed with 1 µm spherical MPs, the differences were not statistically significant. In contrast, *IL-17f3* was significantly downregulated only in response to 1 µm spherical MPs (*P*< .05).

## Discussion

MP and NP pollution represents an emerging threat to organismal health and is ubiquitously prevalent in aquatic ecosystems.^30^, ^31^ Fish, often acting as top predators in these environments, are highly susceptible to the exposure and accumulation of MP and NP contaminants.^32^ Field sampling has confirmed the presence of various fibrous, spherical, and fragmented MPs within the gastrointestinal tract and gills, as well as in the muscle and liver of fish. Furthermore, NPs have been shown to penetrate blood vessels and can even be transmitted to subsequent generations.^33^ While MPs can enter developing fish through surface adhesion or reach internal organs in adults via intestinal absorption or epidermal infiltration, NPs possess the ability to not only penetrate the blastopores of embryos but also reach internal organs in adults through systemic blood circulation.^32^, ^34^

Recent reports demonstrated that NPs induce the disruption of endocytosis, phosphate and calcium metabolism, and lysosomal processes at the cellular level.^25^, ^26^ Furthermore, MP and NP exposure has been shown to disrupt lipid metabolism by activating lipogenic transcription factors or promoting lipid accumulation in hepatic cell lines.^35^, ^36^ However, despite the continuous generation of plastic waste of various shapes and sizes, data regarding their effects at the fish cellular level remain limited compared to studies on individual fish. In this study, the size of MPs and NPs, rather than their shape, was the primary factor affecting cell viability. However, this toxicity was not linearly dependent on size; rather, exposure to larger particles (1 μm spherical MPs) resulted in a significantly greater decrease in cell viability compared to smaller particles (0.2 μm spherical NPs). Additionally, decreased cell viability at relatively high concentrations was more pronounced in marine fish cells (PMF) than in freshwater fish cells (FHM). In PMF cells, long-term exposure (2 weeks) clearly reduced cell survival following treatment with 0.2 and 1 μm spherical MPs/NPs (Supplementary Figure 1). Cellular uptake was also more prominent following exposure to 1 μm spherical MPs than to 0.2 μm spherical NPs at 6, 24, and 48 h. The intensity of cellular uptake was generally stronger in PMF cells than in FHM cells. This difference may be attributed to the fact that while small-sized NPs (0.2 μm) can enter cells, they may also be readily eliminated, whereas 1 μm spherical MPs are likely retained within the cells, leading to greater accumulation.^37^ This size-dependent intracellular accumulation can be explained by differential uptake and elimination mechanisms. Studies have suggested that smaller particles (∼0.2 μm) are subject to active exocytosis, whereas 1 μm particles tend to be sequestered within lysosomes, where they accumulate without being effectively excreted.^37^, ^38^

The toxicity of MP and NP exposure triggers biological defense mechanisms at both molecular and cellular levels. ROS, acting as pivotal signaling molecules, modulate various physiological processes, including inflammation.^30^, ^39^ Oxidative stress resulting from ROS generation can induce apoptosis via both intrinsic and extrinsic pathways.^40^ Previous studies have shown that NP exposure induces ROS production, inflammatory responses, immunotoxicity, DNA damage, and genotoxicity in fish.^19^ In contrast, some reports indicated that ROS production was not detected following exposure to NPs (0.05, 0.5, and 1 μm), despite the observation of inhibited cell proliferation in fish skin fibroblasts.^26^ Similarly, NPs (0.2 and 1 μm) did not significantly trigger cellular ROS in skin cells.^41^ In this study, MP and NP exposure did not induce high levels of ROS in fish fin (PMF) and muscle (FHM) cells, although a significant increase was observed following exposure to 1 μm spherical MPs in both cell lines. Furthermore, the expression of antioxidant enzymes (*SOD*, *CAT*, and *GST*) did not change significantly under both MP and NP exposure. However, the expression of the *NRF2* gene, an early-response transcription factor, was significantly upregulated. Exposure to MPs and NPs appears to induce rapid *NRF2* stabilization and subsequent nuclear translocation as an immediate primary signal. Nevertheless, the transcriptional activation of downstream effectors, such as *SOD* and *CAT*, operates on a different kinetic timescale. Thus, while acute exposure time points effectively reflect early *NRF2* signaling, they may not coincide with the peak expression of subsequent antioxidant defenses.^42^, ^43^

Moreover, pro-inflammatory cytokines, specifically *IL-1β* and *TNF-α*, were significantly induced by 1 µm MPs and 0.2 µm NPs in the present study. These cytokines function as key regulators of immune defense, inflammatory responses, cellular damage, and apoptosis. These findings support previous studies in which exposure to spherical NPs altered the expression of immune-related genes (*IL-1β*, *IL-8*, and *TNF-α*) in the liver tissue of common carp (*Cyprinus carpio*).^44^ In FHM cells, ROS levels were slightly elevated and antioxidant genes remained generally inactive, yet pro-inflammatory cytokines were upregulated following MP and NP exposure. This result suggests that these exposures trigger inflammation through physical signaling or receptor-mediated mechanisms rather than typical chemically induced oxidative stress. This phenomenon may occur when cells attempt to internalize non-degradable MPs. While ROS are primarily generated in large quantities through mitochondrial damage or enzymatic reactions, physical interactions—such as particles piercing intracellular organelles or becoming sequestered in membranes—can initiate signaling without a rapid chemical ROS burst.^45^ Furthermore, when plastics enter a cellular environment, they can bind to surrounding proteins to form a "biomolecular corona."^46^, ^47^ If this protein corona recruits inflammatory proteins or specific signaling molecules, the cell may respond to the ligands adsorbed onto the particle surface rather than the plastic core itself.^46^, ^48^ This allows cells to prioritize an immune response mediated by surface-bound proteins, leading to cytokine release before a significant increase in oxidative stress.^48^, ^49^ As these observations may reflect the toxic effects of acute exposure, further studies are warranted to investigate cell viability, ROS production, and cytokine responses in fish cells following chronic (long-term) exposure.

## CRediT authorship contribution statement

**Kiyun Park:** Writing – review & editing, Writing – original draft, Visualization, Validation, Software, Resources, Methodology, Investigation, Formal analysis, Data curation, Conceptualization. **Trang Thi Nguyen:** Visualization, Validation, Resources, Methodology, Investigation, Formal analysis, Data curation. **Ihn-Sil Kwak:** Writing – review & editing, Writing – original draft, Validation, Supervision, Resources, Project administration, Investigation, Funding acquisition, Data curation, Conceptualization.

## Declaration of Competing Interest

The authors declare that they have no known competing financial interests or personal relationships that could have appeared to influence the work reported in this paper.

## Data Availability

Data will be made available on request. Fisheries Science InstitutePMFandFHMdata

## Footnotes

Appendix A Supplementary data associated with this article can be found in the online version at doi:10.1016/j.cstres.2026.100195.
