# Supplementary material for: Size-dependent internalization of micro- and nanoplastics induces pro-inflammatory and oxidative stress responses in marine and freshwater fish cell lines
Source: Cell Stress Chaperones. 2026 Jul 8;31(4):100195. doi: 10.1016/j.cstres.2026.100195 (PMC13400221; doi:10.1016/j.cstres.2026.100195)
Supplement: Supplementary file 1 — Supplementary material [file mmc1.docx]

**Supplementary Fig. 1.** Cell viability to MP (1 µm) and NP (0.2 µm) exposures on the PMF cell for long-term exposure (2 weeks).


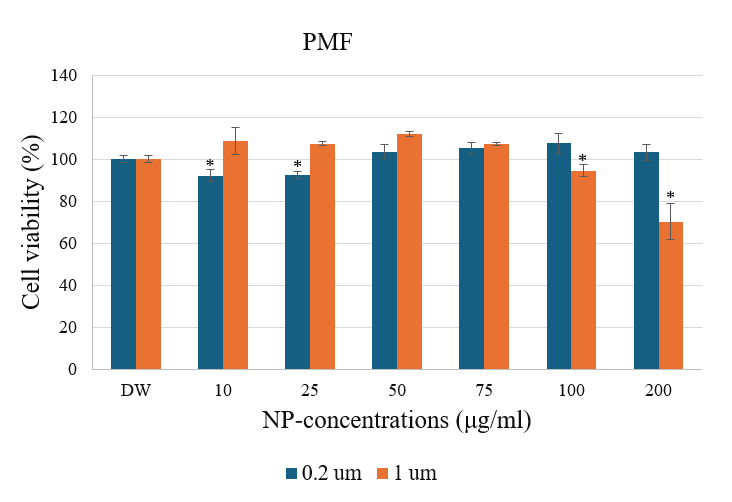


**Supplementary Fig. 2.** Cellular uptake to the spherical MP (6.28 µm) exposure on the PMF and FHM for 48 and 72 h.


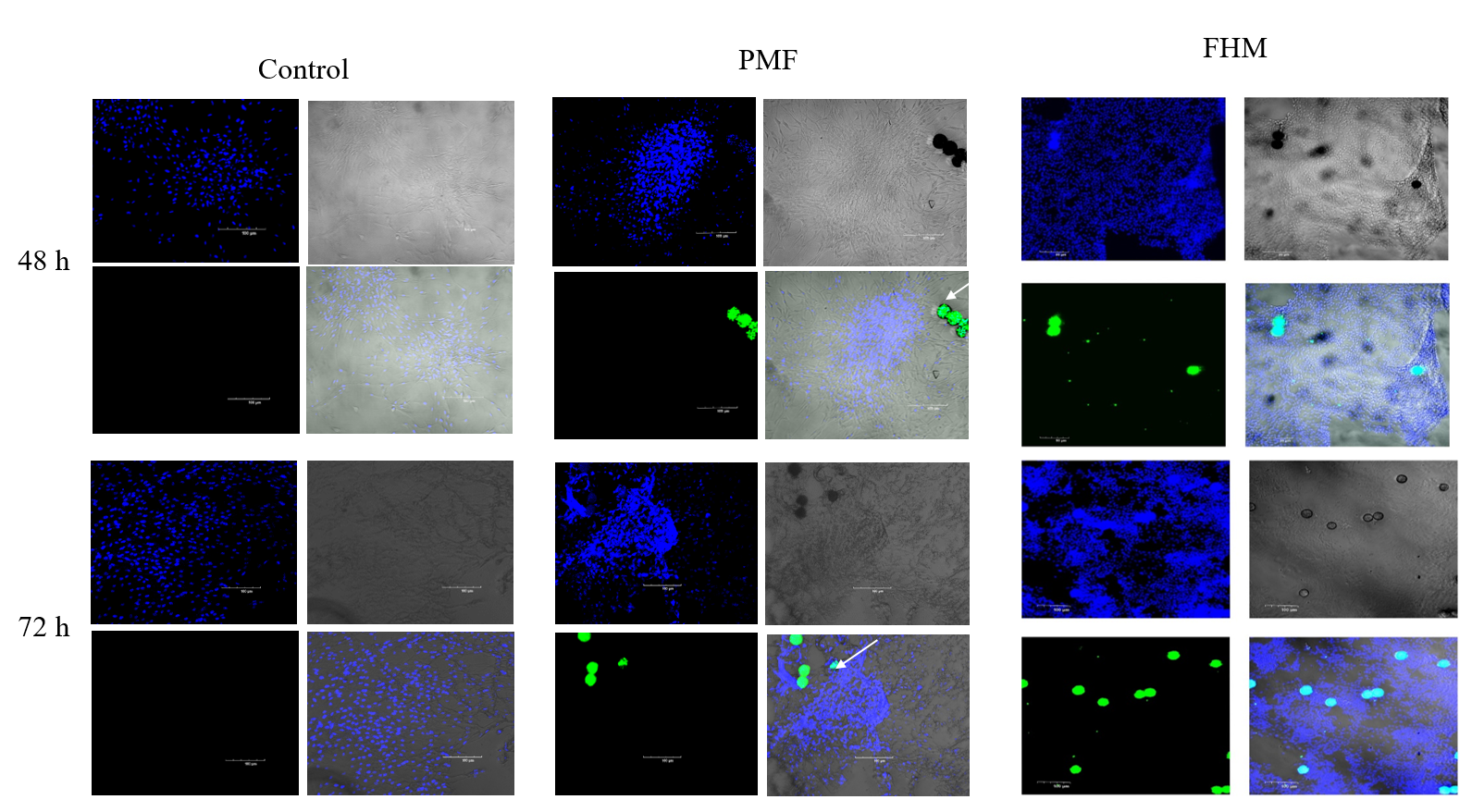


**Supplementary Fig. 3.** Quantitation in the internalization of MP (1 µm) and NP (0.2 µm) on PMF and FHM cells.


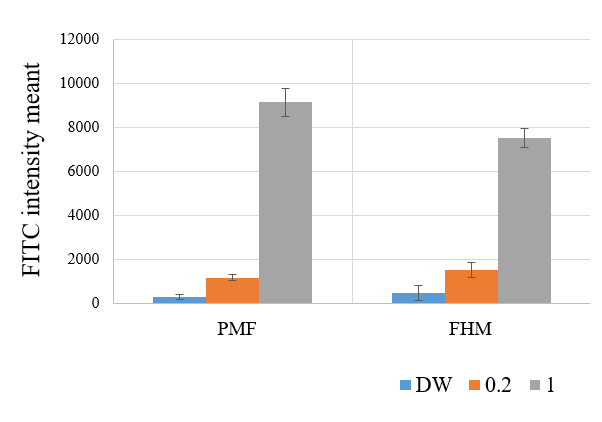


**Supplementary Table 1.** Primer sequences used for mRNA expression analysis

| Gene ID | Gene name | Forward primer | Reverse primer |
| --- | --- | --- | --- |
| [120495043](https://www.ncbi.nlm.nih.gov/gene/120495043) | *GAPDH* | GGATTGTCGTTCCTCCATCTT | ACCCGAACTCATTGTCATACC |
| 120459756 | *NRF2* | AGAGATGAAGCAGCAGTTGAG | TATTCGTTGGGCGAGAAAGG |
| [120476487](https://www.ncbi.nlm.nih.gov/gene/120476487) | *SOD1* | GAAGGAGGATGACTTGGGTAAG | TCTACTCCGTGATGCCAATAAC |
| [120494042](https://www.ncbi.nlm.nih.gov/gene/120494042) | *SOD2* | TACAGGTCTCGTCCCACTAC | TCTCCCAGCTCACAACATTC |
| [120465393](https://www.ncbi.nlm.nih.gov/gene/120465393) | *GSTP-1* | TCAATGGCAATGGCAAACAG | ATCACAGAGCACCAGTTAAGG |
| [120476548](https://www.ncbi.nlm.nih.gov/gene/120476548) | *IFN-G1* | CATCACCAGGACACGAGAAC | CAGGTGTTCGCTGAAGTTTG |
| [120483438](https://www.ncbi.nlm.nih.gov/gene/120483438) | *IL-6* | AGTGTCCTGACGTGGTATAAAG | TTAATGTCCTCCAGCAGTCG |
| [120485191](https://www.ncbi.nlm.nih.gov/gene/120485191) | *TNF-a* | GGAGCATCTGCATACATCTTT | CCTTCTTCAAGACCCAACAAT |
| 120477761 | *CAT* | TAAGGTCTGGTCCCATAAGGA | CTCAACCTCAGCGAAATAGTT |
| 120483092 | *COL2A1A* | CATCTGGTTCTTCTGGTGACC | CCATCAGATCCAGGATTACCCT |
| 120483159 | IL1b | TCCCAGACCAATCTCTAC | CAGGAGGTTGTCATTCTG |
| 120480520 | IL10 | GGCACTAATGGGATGTTG | CAGTGTGTGGTTGATCTG |
| 120480118 | IL17a/f1 | GCACAACTACTCCTTTCG | GAATACCAGCGACATCAG |
| 120494156 | IL17a/f3 | GGTCTATGTCAGGGAGTTC | TATGGGCTGGTGAGAAAG |
| 120461073 | IL4 | CAGCATCACATCCTCTACAC | TGGGTCCATGTTGGTTTC |
